# Circulating extracellular vesicles exhibit a differential miRNA profile in gestational diabetes mellitus pregnancies

**DOI:** 10.1371/journal.pone.0267564

**Published:** 2022-05-25

**Authors:** Shanthie Thamotharan, Shubhamoy Ghosh, Laura James-Allan, Margarida Y. Y. Lei, Carla Janzen, Sherin U. Devaskar

**Affiliations:** 1 Departments of Pediatrics, David Geffen School of Medicine at UCLA, Los Angeles, California, United States of America; 2 Obstetrics and Gynecology, David Geffen School of Medicine at UCLA, Los Angeles, California, United States of America; University of Mississippi Medical Center, UNITED STATES

## Abstract

We undertook a prospective temporal study collecting blood samples from consenting pregnant women, to test the hypothesis that circulating extracellular vesicles (*EVs*) carrying specific non-coding *microRNA* signatures can underlie gestational diabetes mellitus (GDM). To test this hypothesis, *miRNA* cargo of isolated and characterized *EVs* revealed contributions from the placenta and differential expression at all three trimesters and at delivery between pregnant and non-pregnant states. Many *miRNAs* originate from the placental-specific chromosome 19 microRNA cluster (19MC) and chromosome 14 microRNA cluster (14MC). Further a positive correlation emerged between third trimester and at delivery *EVs* containing *miRNAs* and those expressed by the corresponding post-parturient placentas (*R value* = 0.63 to 0.69, p value = 2.2X10^-16^), in normal and GDM. In addition, distinct differences at all trimesters emerged between women who subsequently developed GDM. Analysis by logistic regression with leave-one-out-cross validation revealed the optimal combination of *miRNAs* using all the circulating *miRNAs* (*miR-92a-3p*, *miR-192-5p*, *miR-451a*, *miR-122-5p*), or using only the differentially expressed *miRNAs* (has-miR-92a-3p, hsa-miR-92b-3p, hsa-miR-100-5p and hsa-miR-125a-3p) in GDM during the first trimester. As an initial step, both sets of *miRNAs* demonstrated a predictive probability with an area under the curve of 0.95 to 0.96. These *miRNAs* targeted genes involved in cell metabolism, proliferation and immune tolerance. In particular genes of the *P-I-3-Kinase*, *FOXO*, insulin signaling and glucogenic pathways were targeted, suggestive of placental connectivity with various maternal organs/cells, altering physiology along with pathogenic mechanisms underlying the subsequent development of GDM. We conclude that circulating *EVs* originating from the placenta with their *miRNA* cargo communicate and regulate signaling pathways in maternal organs, thereby predetermining development of GDM.

## Introduction

As pregnancy complications continue to increase worldwide, there have been increasing efforts to study the first-trimester as a window of opportunity for early identification, prediction of, and the optimal timeframe for implementation of measures to prevent multifactorial maternal disease [1]. Gestational diabetes mellitus (GDM) represents the most common disorder of pregnancy with a growing incidence of obesity and diabetes mellitus in the general population. Specifically, GDM is the most common metabolic disorder in pregnancy, defined as impaired glucose tolerance first diagnosed during mid-gestation and is associated with peripheral maternal insulin resistance, maternal inflammation, and placental dysfunction.

Adverse pregnancy outcomes confer increased risk for complications during pregnancy for both the mother and child. GDM causes substantial maternal morbidity and mortality leading to increased risk of cardiovascular disease later in life for the mother [2]. Additionally, infants born to mothers who have developed adverse outcomes during their pregnancies are at risk of short- and long-term complications, such as obesity, diabetes, and cardiovascular disease later in life [3, 4]. Consequently, there is a need to elucidate the pathophysiology of these complications of pregnancy and improve diagnostic testing so that diagnosis and subsequent intervention can occur earlier in pregnancy. In doing so, improving maternal and fetal health during gestation and subsequently throughout life.

Recent studies have shown that extracellular vesicles (EVs) play a key role in pregnancy [5, 6] and in complications of pregnancy, including GDM [7, 8] and the associated preeclampsia (PE) [9]. EVs are nanosized particles with heterogenous content, including bioactive proteins, microRNAs (miRNAs), mRNAs and lipids [5]. Their lipid bilayer allows stable transport of content to recipient cells, allowing intercellular communication. EVs are classified into three main subtypes, exosomes, microvesicles and apoptotic bodies, based on their size and mechanism of formation and release. Due to lack of specificity of EV markers and overlap in size between these subgroups, guidelines [10] have recommended the classification of EVs dependent on a physical characteristic such as their size; consequently EVs that range in size up to or around 100nm, which will encompass both exosomes and microvesicles, are termed small extracellular vesicles (sEVs).

The placenta releases EVs into the maternal circulation from 6 weeks of gestation and the number of total and placental-specific EVs increase across gestation, peaking in the third trimester and decreasing after delivery [5, 11]. Additionally, the concentration of EVs is further increased across gestation in pregnancies diagnosed with GDM [12]. Circulating EVs in these conditions have a unique cargo, including differentially expressed miRNAs [13–15] which are thought to play a role in their disordered pathophysiology [7, 16]. Therefore, there is scope for EVs in the maternal circulation to be utilized as markers of placental and fetal health during pregnancy. However, there is a lack of comprehensive data investigating the miRNA signature of EVs in a range of adverse pregnancy outcomes throughout gestation.

Consequently, in our prospective pregnancy study our aim was to elucidate the miRNA content of circulating plasma EVs across gestation in women subsequently diagnosed with GDM, and compare these to unaffected pregnancies. Pregnant women were enrolled in the first trimester and followed temporally throughout pregnancy. We hypothesized that the miRNA cargo of circulating EVs will differ across the first, second and third trimesters of pregnancy in parallel to the differing needs and development of the fetus and that the miRNA profile of EVs isolated from women with healthy pregnancies will differ from those complicated by GDM across gestation.

## Materials and methods

### Collection of blood and placenta samples

“PARENTs” is a prospective cohort study, approved by the UCLA Institutional Review Board (IRB:15–001388), in which pregnant women were recruited in the first trimester of pregnancy (clinicaltrials.gov: #NCT02786420). Before 11 weeks of gestation written informed consent was obtained and women were seen at three subsequent study visits during pregnancy and at delivery. Maternal blood samples were collected from non-pregnant women and pregnant women in the first trimester (11–14 weeks), second trimester (19–22 weeks), third trimester (36 weeks) and at delivery. Placenta samples were collected at delivery from singleton pregnancies after 36 weeks of gestation. We divided the subjects into healthy pregnancies (n = 7) or pregnancies diagnosed with GDM (n = 14). Blood was collected from non-pregnant women (n = 3) to serve as controls and a baseline for the pregnancy samples. The clinical grouping of GDM versus normal pregnancies was achieved based on established criteria as previously reported [17].

### Isolation of EVs from maternal plasma

Maternal peripheral venous blood was collected into EDTA-coated tubes and centrifuged at 3200xg for 7 min, to separate the plasma fraction, which was stored at -80°C for future use. Extracellular vesicles (EVs) were isolated from 1ml of plasma by using polymer-based precipitation (EXOQ5TM-1, System Biosciences, Mountain View, CA, USA) by following the manufacturer’s protocol. Briefly, plasma samples were centrifuged at 3000xg for 15 minutes to remove cells and the rest of the cellular debris. The supernatant was collected, incubated with thrombin (final concentration of 5U/ml) for 5 minutes at room temperature to catalyze the conversion of fibrinogen to fibrin which precipitated, and was centrifuged off at 10,000 rpm for 5 minutes. The supernatant, containing EVs, was incubated with ExoQuick exosome precipitation solution for 1h at 4°C (pre-optimized conditions). This incubated solution was centrifuged at 1500xg for 30 minutes, and the obtained supernatant was aspirated and saved for analysis (serving as the negative control), and the pellet containing the precipitated EVs was resuspended in PBS.

### Immunoblotting

Equal amount of protein in an equal volume of each sample was mixed with Laemmli’s buffer and separated by SDS-PAGE. Proteins were transferred to nitrocellulose membranes overnight at 4°C. Following transfer, membranes were blocked with 5% (w/v) milk in PBS-T. Membranes were incubated overnight with primary antibodies: CD63 [1:1000] (EXOAB-CD63A, System Bioscience, Palo Alto, CA), PLAP [1:750] (Abcam, Cambridge, MA), Calnexin [1:1000] and Flotillin [1:750] (Cell Signaling Technology Danvers, MA). HepG2 cell (ATCC) lysate was used as the–ve control for the PLAP antibody and +ve control for the Calnexin antibody. Membranes were then incubated with the appropriate peroxidase labeled secondary antibodies. Protein bands were visualized with chemiluminescence using the ChemiDoc Imaging System (Bio-Rad Laboratories, Hercules, CA).

### Transmission electron microscopy (TEM)

Isolated EV pellets were fixed in 2% (v/v) paraformaldehyde. Fixed EVs were then applied to a Formvar-carbon coated grid and incubated with 1% glutaraldehyde. The samples were first contrasted in uranyl-oxalate solution, with pH 7 and then contrasted and embedded in 4% uranyl acetate and 2% methyl cellulose. The samples were examined in a JEOL 100CX transmission electron microscope.

### Dynamic light scattering

Dynamic light scattering (DLS) was used to detect the size of the isolated vesicles. Isolated EVs were placed in a microcuvette and analyzed using a Zetasizer Nano Instrument (Malvern Instruments Ltd, UK) at room temperature.

### Flow cytometry

Streptavidin coated beads (Exo-Flow exosome purification kit) were conjugated with biotinylated PLAP antibody (NB110 – 3638N, Novus Biologicals, Englewood, CO) and antibody coupled beads were used to capture EVs isolated by ExoQuick exosome isolation kit. After washing off the unbound EVs, captured EVs were stained with reversible Exo-FITC before FACS analysis.

### Placental tissue preparation

Fresh placentas at delivery were collected as soon after expulsion as possible. Full thickness section was taken closer to central two thirds of the placenta, cut into 0.5 -1cm width containing membranes. Using sterile technique, a triangular full thickness segment of the placenta, with its convex base at the lateral edge of the placenta and its apex at the placental center near the cord insertion site, was obtained. Both the decidual layer along the basal plate as well as the chorionic surface and membranes were removed by sharp dissection and a representative placental fragment (0.5–1 cm width) was obtained in the middle of the initial placental depth, approximately 10 mm from the basal and chorionic plates, and used for RNA extraction. The tissue was washed in ice-cold 1 X phosphate-buffered saline (PBS) to remove maternal blood. Two sets of samples were snap-frozen in liquid nitrogen and stored at -80°C for subsequent RNA extraction [18].

### RNA extraction from placenta and EVs

Total RNA was extracted from isolated EVs using miRNeasy Mini Kit (Qiagen, Valencia, CA) following the manufacturer’s instructions, with minor modifications. Total RNA from placental tissue was isolated using Zymo Direct-zol RNA miniprep kit (#R2050; Zymo research, Irvine, CA, USA) following manufacturer’s instructions. Briefly, the snap frozen and subsequently powdered tissue was incubated in TRIzol (#15596026; ThermoFisher Scientific, Waltham, MA, USA) for 5 min. The mixture was homogenized with a hand-held homogenizer for 1 min on ice and centrifuged at high speed for 2 min at 4°C. Collected supernatant was directly applied to Zymo spin column and centrifuged at high speed to bind RNA to silica membranes. On-column digestion of genomic DNA was performed using DNaseI (supplied with the kit) followed by washing with ethanol containing wash buffer. RNA was eluted with 10mM Tris and the concentration was measured using Qubit3 Fluorometer (ThermoFisher Scientific, Waltham, MA, USA). The Agilent Bioanalyzer or Tape Station (Agilent, Santa Clara, CA, USA) was used to determine RNA integrity numbers (RIN) prior to library preparation.

### Preparation and sequencing libraries

For *miRNA* sequencing libraries total RNA (6 μl) extracted from EVs was used. Libraries were prepared by NEB Next Multiplex small RNA Library prep kit (NEB E7300S; New England Biolabs, Inc, Ipswich, MA, USA) following manufacturer’s instructions. Placenta miRNA libraries were prepared using TruSeq Small RNA Library prep kit–Set A RS-200-0012 and Set B RS-200-0024, according to the manufacturer’s instructions. These libraries were subjected to sequencing employing HiSequencer. miRNA libraries were sequenced using HiSeq-2500 with single-end 50bp read (Illumina Inc.; San Diego, CA, USA).

### Analysis of miRNA seq library

We used miRDeep2 package to align sequencing reads to known human miRNAs [19] and for quantification. For aligning, we input the raw sequencing files into the script mapper.pl (a component of miRDeep2). This script trimmed the adapter sequence AGATCGGAAGAGCACACGTCTGAACTCCAGTCAC from libraries, discarded reads with fewer than 18 nucleotides, and aligned the reads to the genome (hg38) using the Bowtie software [20]. We applied options -e, -h, -i, -j, -k, -l 18, and -m. Genome-aligned reads were matched to known human miRNAs from miRBase version 21 [21] and quantified using the script quantifier.pl (a component of miRDeep2) using options–d and–t hsa. We allowed one mismatch and included 2 nucleotides upstream and 5 nucleotides downstream of the mature sequence. Raw read counts of individual samples were normalized for sequencing depth and RNA composition and differentially expressed genes were determined by the DESeq2 software [22]. A log 2-fold-change ≥ 1 or ≤ −1 with a false discovery rate (FDR) < 0.05 was selected to determine inter-group differential expression/abundance. The FDR was calculated in DESeq2 using the Benjamin-Hochberg correction [22].

We have employed MIENTURNET (MicroRNA ENrichment TURned NETwork), an interactive web-based tool, for microRNA-target enrichment analysis [23]. We used a p-value cut-off of < 0.05 to determine significantly abundant miRNAs in first trimester samples from GDM women compared to normal pregnancies. The demultiplexed fastq files and expression matrix are available at GEO (GSE186883).

### Predictive model for early biomarker discovery

EV miRNAs detected in the first trimester of pregnancy were used to develop a predictive model for early biomarker determination. We built a logistic regression (LR) model with elastic-net regularization, that has been trained using a leave-one-out cross validation (LOOCV), and selected the model containing corresponding miRNAs that was able to detect >80% of the true positive values.

### Statistical analysis

Statistical analysis was performed using DESEq2 package from R. All data were log transformed. Temporal comparisons between the two groups were performed employing the Kruskal-Wallis one-way analysis of variance followed by Dunn’s post-hoc test, and when multiple comparisons were performed, Bonferroni’s correction was performed.

## Results

### Clinical characteristics

A total of 24 women were included in the study, 3 healthy non-pregnant women to serve as baseline controls for the pregnant state, 7 women with healthy normal pregnancies, and 14 women with GDM. Maternal demographics and fetal data are detailed in Table 1. While no change in HbA1c between groups was observed in the first trimester of pregnancy ruling out pre-existing diabetes mellitus, the OGTT-one hour screen revealed higher blood glucose concentrations in GDM versus the normal pregnancies (p = 0.0004). There were no significant differences in pre-pregnancy BMI, gestational age at delivery or birth weight between the two groups (Table 1).

**Table 1:** Clinical characteristics.

	NORMAL	GDM
Number of samples	7	14
Ethnicity	Caucasian = 3	Caucasian = 6
	Asian = 2	Asian = 4
	Others = 2	Others = 4
Pre-Pregnancy or pregnancy BMI at 1^st^ prenatal visit	25.12±1.49 (N = 7)	24.53±1.24 (N = 14)
Glucose (mg/dl)- GTT 1hr screen	113.43±10.33 (N = 7)	179.78±9.55 (N = 9)*
Blood pressure Systolic (mm Hg)	120.14 ±6.21(N = 7)	123.23±3.48 (N = 13)
Blood pressure Diastolic (mm Hg)	70.29±3.27 (N = 7)	74.69±2.44 (N = 13)
Placental Wt.(g)	483.53±20.27 (N = 7)	487.17±19.15 (N = 12)
Birth Wt. (percentiles)	63.5±7.48 (N = 7)	55.41±6.57 (N = 12)
Gestational Age (weeks)	39.10±0.52 (N = 7)	38.48±0.34 (N = 13)
Baby Sex (M/F)	M = 2, F = 5	M = 8, F = 6

* p-value = 0.0004 Data presented as mean ± SEM. Sample size shown in parenthesis.

### Characterization of the isolated EVs

**Fig 1:**
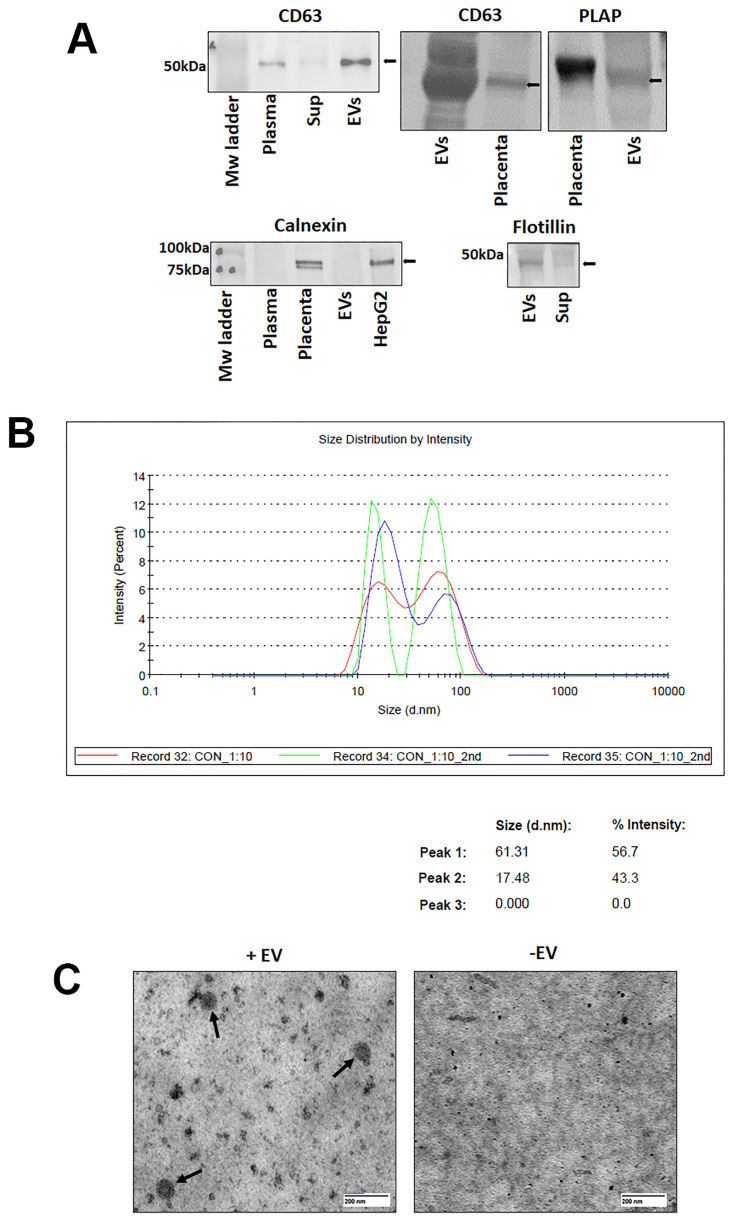
Characterization of plasma EVs (EVs). **A.** Immunoblot showing enrichment of CD63 (~60 kDA) in EVs compared to plasma, supernatant (Sup), and placenta and enrichment of placental-specific placental alkaline phosphatase (PLAP; ~70 kDA) (top panel). Immunoblot showing absence of cell-specific marker Calnexin (~90 kDA) in EV and plasma samples and presence in placenta and HepG2 cell line (bottom left panel) and enrichment of flotillin, an EV-specific marker in EVs compared to plasma. **B.** Size distribution and intensity of EVs detected by dynamic light scattering (red, green, blue peaks represent replicates) with table below showing the average size and intensity. **C.** Representative transmission electron microscopy of EVs (+EVs) with a negative control (-EVs), scale bar = 200 nm. Black arrows demonstrate the EVs.

Immunoblotting of plasma and EVs showed an enrichment of EV specific marker CD63 in precipitated EVs over the corresponding supernatant, whole plasma alone, and placenta. In addition, presence of PLAP supported the contribution to these EVs from placental trophoblasts (Fig 1A). EVs were further characterized by dynamic light scattering (DLS) and TEM. Average size of EVs as determined by DLS was 17-61nm with an intensity of 43–56% respectively. (Fig 1B). TEM analysis revealed that EVs displayed a circular morphology. (Fig 1C). Subsequent analysis with Flow cytometry ensured presence of PLAP positive EVs among our isolates (S1 Fig).

### miRNA profile in total plasma EVs

**Fig 2:**
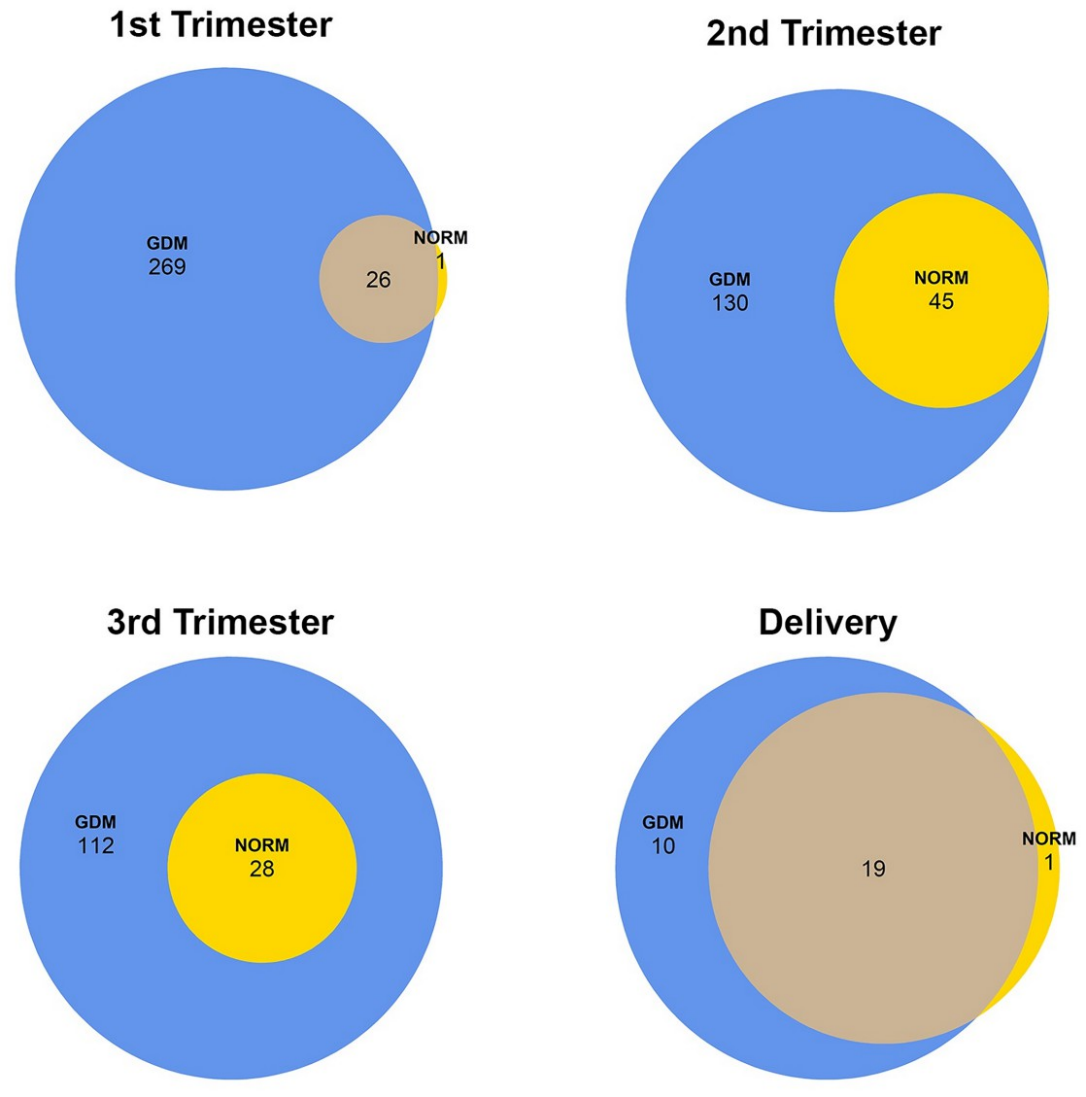
Differentially abundant EV-associated miRNAs at three trimesters of gestation and delivery in normal and gestational diabetes mellitus (GDM) pregnancies. Venn diagrams display the number of differentially abundant miRNAs in EVs isolated from normal pregnant (NORM) or gestational diabetes mellitus (GDM) samples compared to non-pregnant (NP) samples in (A) first trimester, (B) second trimester, (C) third trimester and (D) at delivery. Yellow represents Normal pregnancy and blue GDM. All represented genes had a log2 fold-change ≥ 1 or ≤ −1 and FDR < 0.05. The FDR was calculated in DESeq2 using the Benjamin-Hochberg correction [22].

miRNA sequencing identified pregnancy associated differentially abundant EV-containing miRNAs of 296 in the first trimester, 175 in the second trimester, 140 in the third trimester and 30 at delivery (Fig 2), compared to the non-pregnant control. EVs isolated from GDM pregnancies had 269 and 130 uniquely abundant miRNAs during the first and second trimesters, respectively. Whereas only 112 and 10 miRNAs were uniquely identified during the third trimester and at delivery, respectively, in GDM pregnancies (Fig 2).

**Fig 3:**
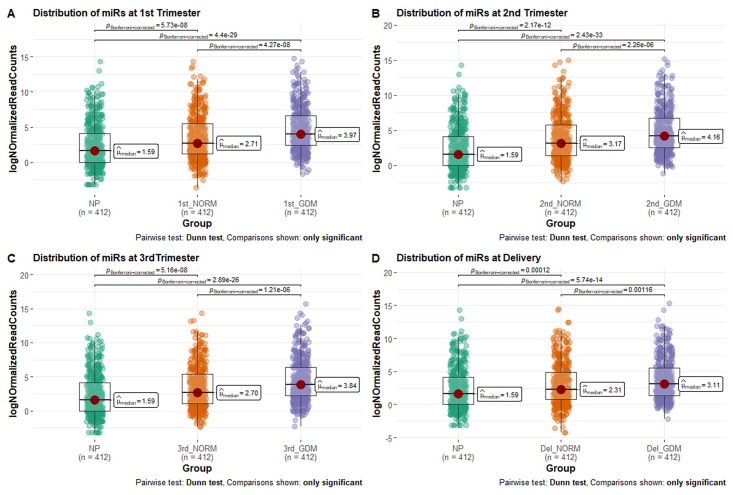
Distribution of EV derived miRNAs at three trimesters of gestation and at delivery in normal and gestational diabetes mellitus (GDM). Boxplots show the distribution of EV-derived miRNAs in non-pregnant (NP), normal pregnant (NORM) and gestational diabetes (GDM) maternal plasma samples in (A) first trimester, (B) second trimester, (C) third trimester and (D) at delivery. Abundance is shown as Log-normalized read counts. Significance of the difference between groups was calculated using Kruskal–Wallis one-way analysis of variance followed by post-hoc analysis using Dunn’s test. Bonferroni corrected p-values demonstrate significant differences.

We evaluated the distribution of miRNAs from total circulating EVs among the three experimental groups (Fig 3A–3D). There was a significant enrichment of miRNAs in EVs during normal pregnancy as compared to the non-pregnant subjects across gestation (S4 Fig). miRNAs were significantly enriched in total EVs isolated from women with GDM throughout pregnancy compared to normal pregnancy (S4 Fig).

### Placental-specific miRNA profile in plasma EVs

**Fig 4:**
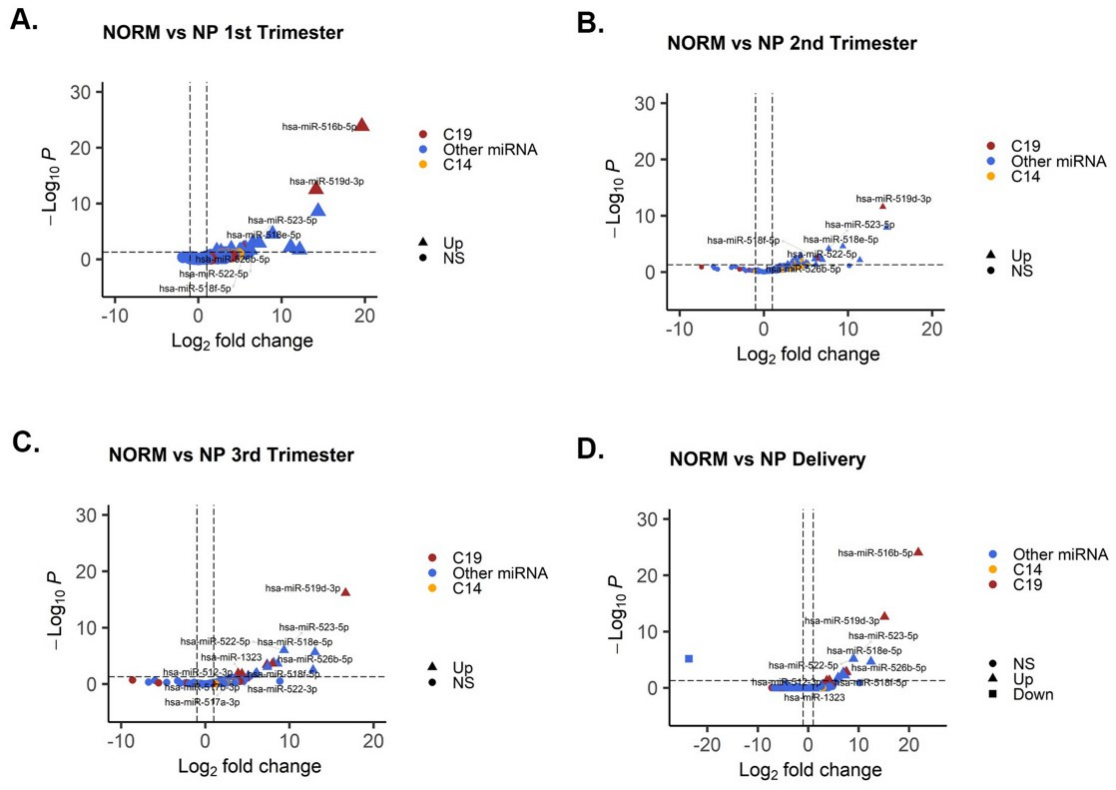
Pregnancy specific EV-derived miRNAs at three trimesters of gestation in normal pregnancy. Volcano plots show differential abundance of pregnancy-specific miRNAs from plasma EVs between non-pregnant (NP) and normal pregnancy (NORM) in **(A)** first, **(B)** second and **(C)** third trimesters of pregnancy and (**D**) at delivery. Filled circles represent no significant difference, triangles represent miRNA with upregulation and squares represent downregulation. Red denoting chromosome 19 microRNA clusters (C19MCs) while yellow denoting chromosome 14 microRNA clusters (C14MCs) and other non-C19MC or non-C14MC miRNAs are represented in blue.

To elucidate and characterize miRNAs enriched in pregnancy, we compared the expression of miRNAs in plasma EVs from normal pregnancy to non-pregnant samples. There was significant enrichment of miRNAs across gestation, including from the chromosome 19 (C19) miRNA cluster (Fig 4A–4D; S1 Table).

**Fig 5:**
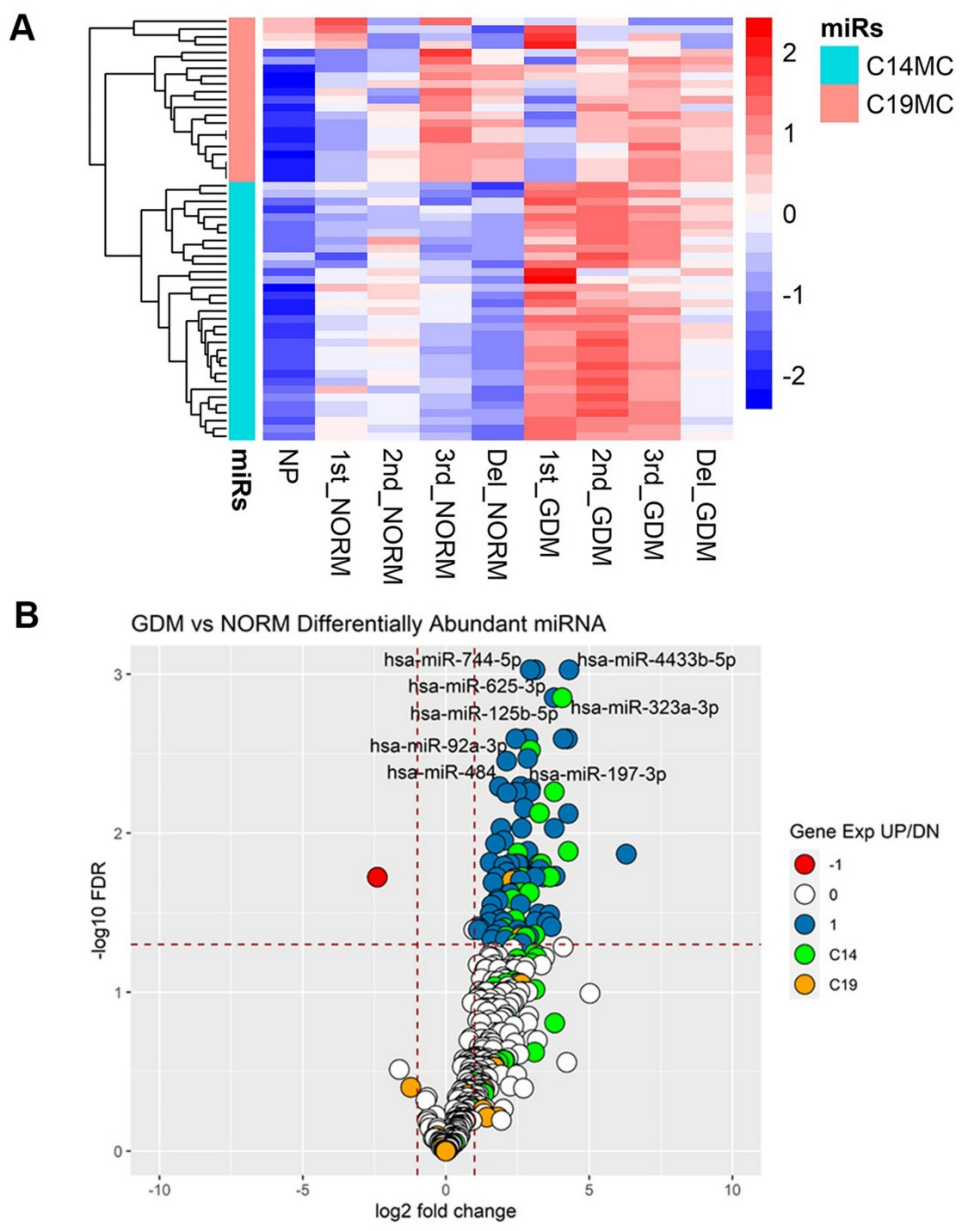
Differential abundance of EV-derived miRNAs in gestational diabetes mellitus (GDM) pregnancies. Heatmap showing expression of placenta-specific C19MC and C14MC in EVs isolated from non-pregnant women (NP), and during 1^st^, 2^nd^, 3^rd^ trimesters and at delivery (del) in normal pregnancy (NORM) and gestational diabetes (GDM) **(A).** Volcano plots display miRNAs from EVs isolated from gestational diabetes mellitus (GDM) **(B)** compared to normal pregnancies, during the first trimester of pregnancy. Red dots represent downregulated miRNAs, white dots represent miRNAs that are not significant for differential expression, blue dots represent upregulated miRNAs, green dots represent miRNAs from the C14MC and orange dots represent miRNAs from the C19MC.

Clustering analysis showed a temporal pattern of plasma EV derived C19MC and C14MC miRNA expression in pregnant women, with an enrichment of C19MC and reduction of C14MC miRNAs with advancing gestation, and a distinct pattern of differential expression between the various subject groups (Fig 5A). In the first trimester, there were 92 differentially abundant miRNAs, 19 from the C14MC and 2 from C19MC, in EVs isolated from GDM pregnancies compared to that from normal pregnancies (Fig 5B, S2 Table).

### Expression pattern of miRNAs from placental tissue

**Fig 6:**
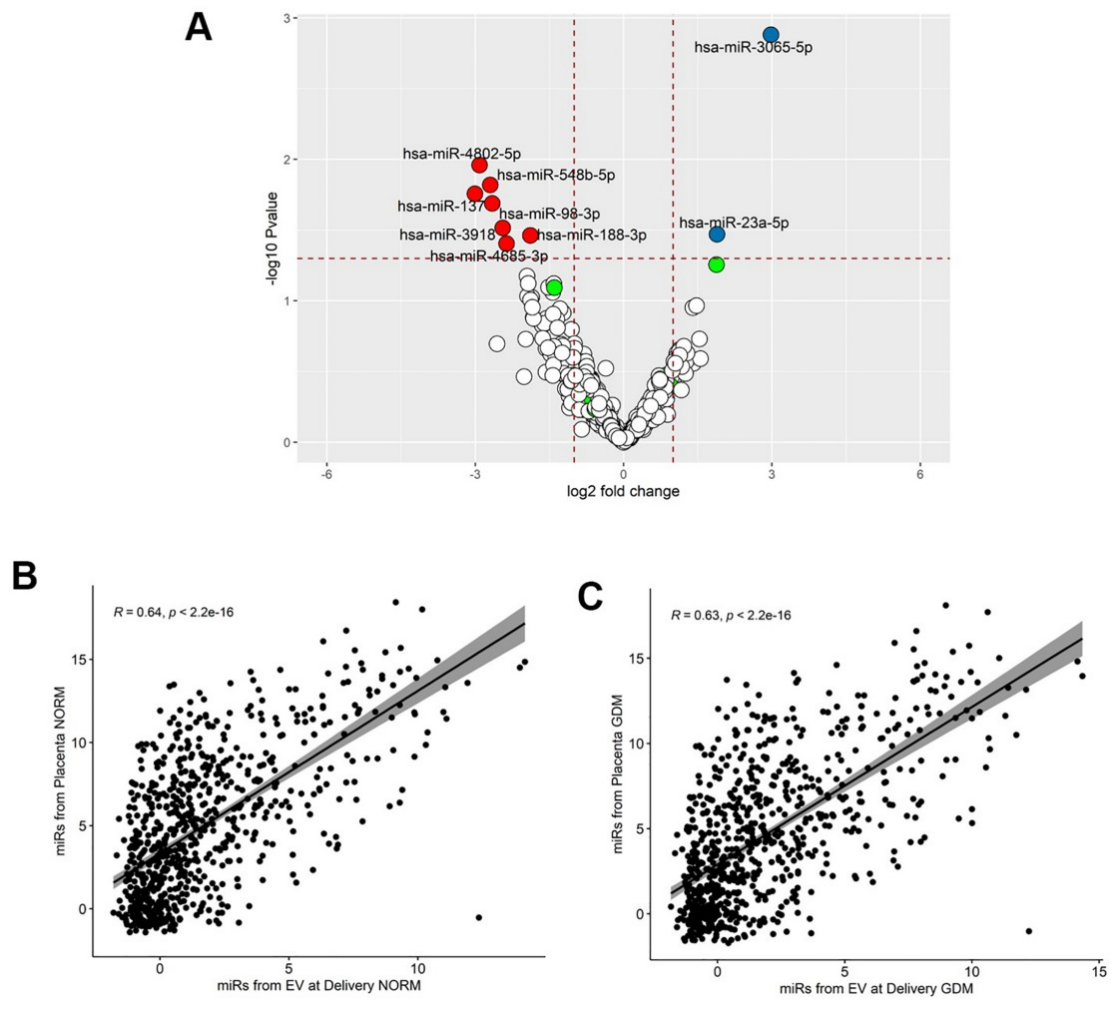
Differential abundance of miRNAs in placental tissue. Volcano plots show expression of miRNAs in term placental tissue from pregnancies complicated by **(A)** GDM compared to normal pregnancies. Correlation between miRNAs from the term placenta and EV-derived miRNAs isolated from maternal plasma at delivery was performed in **(B)** normal pregnancy and **(C)** GDM.

We isolated and sequenced miRNAs collected from placental tissue at term from normal pregnancy and GDM pregnancies. Our analysis showed 9 differentially expressed miRNAs in GDM when compared to normal pregnancy (Fig 6A). No C14 or C19 miRNA clusters were significantly up or downregulated in GDM. We compared the abundance of miRNAs from isolated EVs at delivery to placental tissue and found a positive correlation in normal pregnancy (R = 0.64, p = 2.2 x 10^−16^, Fig 6B) and GDM (R = 0.63, p = 2.2 x 10^−16^, Fig 6C, S3 Table). Similar positive correlations were observed when the abundance of miRNAs from isolated EVs obtained at the third trimester were compared to placental tissues as well (Normal pregnancy: R = 0.66, p < 2.2 X 10^−16^; GDM: R = 0.64, p < 2.2 X 10^−16^; Fig 6B & 6C, S3 Fig).

**Fig 7:**
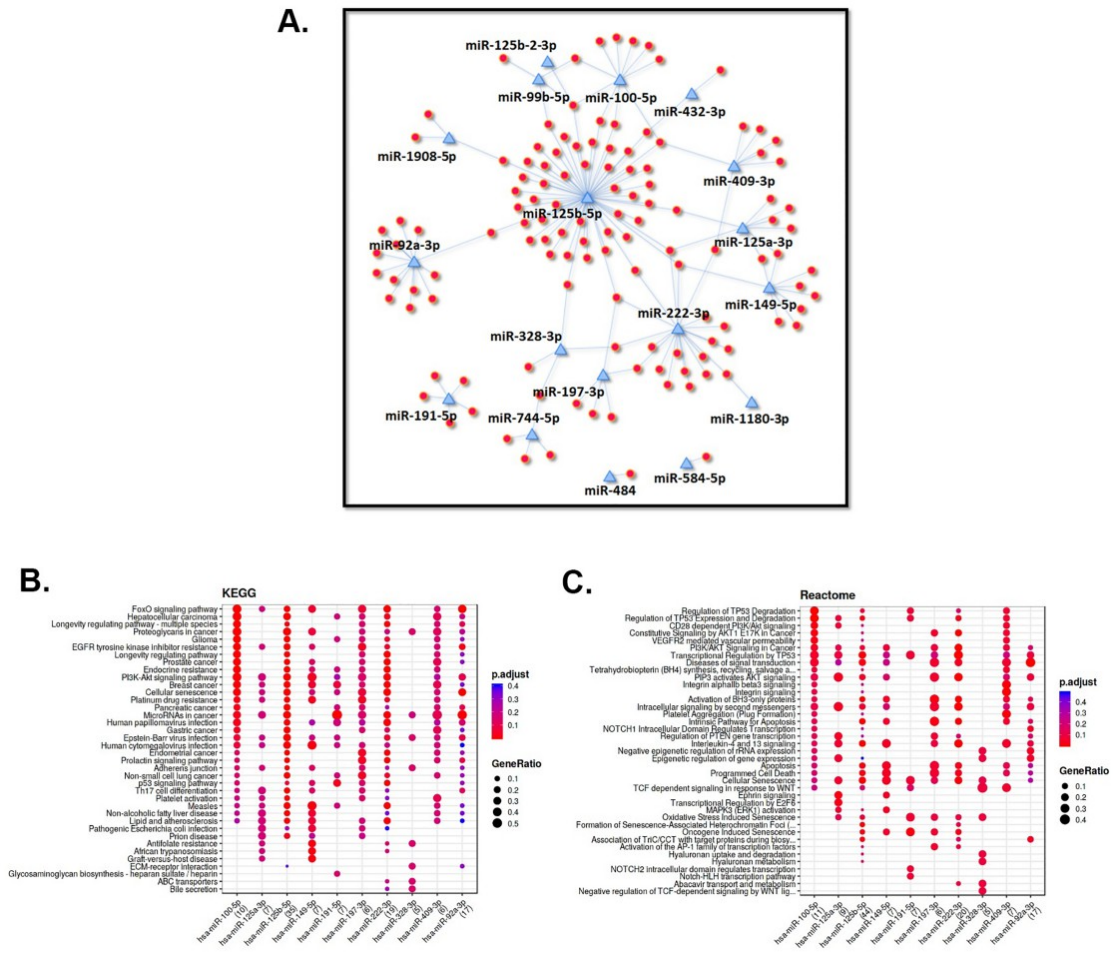
Predicted miRNA-target mRNA interaction in gestational diabetes mellitus (GDM) during early gestation. A). The miRNA-Target mRNA network depicts targets of the top 20 differentially expressed EV-derived miRNAs from GDM in the first trimester of pregnancy. Blue triangles represent miRNAs while red dots refer to their targets. Dot plots represent functional enrichment analysis depicting target genes of selected miRNAs. The Y-axis displays the annotation categories KEGG pathways (**B**) and Reactome pathways (**C**) while the X-axis depicts the selected miRNAs.

Enrichment analysis was performed using the miRNA target genes to interpret the post-transcriptional role of miRNAs in pregnancies with GDM. Using a p value of <0.05 we created a network of miRNA-gene targets from the top 20 differentially abundant miRNAs in EVs isolated from first trimester plasma samples of subjects who went on to develop GDM (Fig 7A, S4 Table). Further analysis with predicted miRNA targets demonstrated enrichment of KEGG (Fig 7B) and reactome (Fig 7C) pathways.

**Fig 8:**
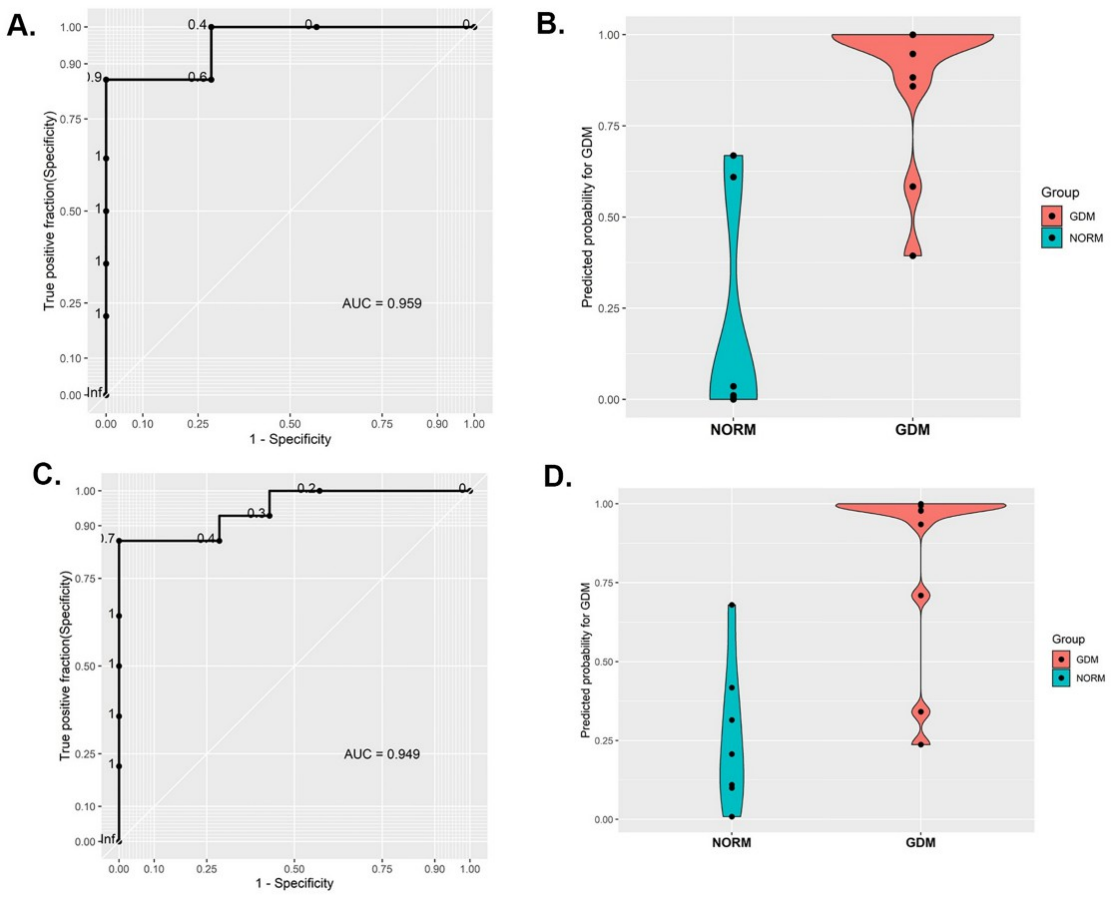
Receiver operator characteristics (ROC) curve for gestational diabetes GDM early gestation prediction. **(A&C)** Receiver operator characteristic (ROC) curve generated using miRNAs isolated from plasma derived EVs of first trimester of pregnancy with GDM (n = 14) versus normal pregnancy (n = 7). (**B&D**) violin plot of predicted probability for GDM with 0 = low probability and 1 = high probability in normal (NORM) and GDM. (**A,B** = approach using all miRNAs detected in GDM, **C,D** = approach using differentially expressed genes alone).

As an initial attempt at determining pregnancy complication of GDM associated transcripts, we focused on the first trimester miRNA expression of EVs. We first employed an approach by pooling all the miRNAs detected in GDM subjects as a group to build a classifier towards predicting GDM as early as in the first trimester of pregnancy. Using elastic-net regularization, we built a logistic regression (LR) model which was trained using a leave-one-out cross validation (LOOCV). We employed all of the isolated EV expressed miRNAs in which the model detected ~90% of the true positive values with an AUC of 0.95 (Fig 8A), thereby demonstrating a probability of developing GDM (Fig 8B). The most recurrently noted miRNAs in the model were hsa-miR-92a-3p, hsa-miR-192-5p, hsa-miR-451a and hsa-miR-122-5p. Simultaneously, we also employed the model including only the differentially expressed/abundant miRNAs between GDM and NORM, which detected ~94% of the true positive values with an AUC of 0.94 (Fig 8C) with a probability of developing GDM (Fig 8D). Most recurrent features in this second analysis were hsa-miR-92a-3p, hsa-miR-92b-3p, hsa-miR-100-5p and hsa-miR-125a-3p. We next employed a previously published cohort of 8 normal and 9 GDM pregnancies [8] as our validation cohort and tested the miRNAs detected from our prediction model on the available 2^nd^ trimester samples alone. In this separate cohort, we detected ~76% true positives with an AUC of 0.764 with 2^nd^ trimester samples as opposed to the 1^st^ trimester samples from our present study.

## Discussion

The results of this study demonstrate that there is a specific profile of miRNAs encapsulated by EVs in the maternal circulation across gestation in GDM, and that EV-associated miRNAs from the first trimester of pregnancy have the potential to act as an early gestation predictor of the subsequent development of GDM, prior to the emergence of characteristic clinical or biochemical features. Our results confirmed that the expression and abundance of EV-associated miRNAs change temporally with advancing gestation in normal pregnancy, and further detected differential expression and abundance of circulating miRNAs in pregnancies with GDM at all phases of gestation. These results suggest that intrauterine environmental perturbations due to gestational metabolic adversities regulate the cargo contained in circulating EVs.

Complications during pregnancy can lead to adverse pregnancy outcomes but also increase the risk of cardiovascular and metabolic diseases in the mother postpartum and later in life for infants born to mothers who endure complications during pregnancy. Currently available diagnosis for these disorders mostly relies on conventional screening or diagnostic methods in the late second and third trimesters. There is a lack of biomarkers for early gestation prediction of the most severe and common complications, including GDM. Biomarkers that can be utilized in early gestation can lead to increased vigilance and monitoring with the need to develop introduction of safe and timely early interventions and treatments, targeting reduction of the disease severity towards improving long-term health complications for mother and offspring. With the development of non-invasive diagnostic capabilities, circulating extracellular vesicles (EVs) in pregnant women have become an attractive candidate for detection and discovery of non-invasive biomarkers, that have the potential of heralding the subsequent development of pregnancy related complications. This is particularly important given the controversy regarding the ineffectiveness on both the mother and baby outcomes of early screening for GDM, employing the currently available algorithms related to high risk clinical features, or single biomarkers derived from metabolomics and proteomics reflecting metabolism or inflammation [24].

In a previous study we examined cell-free DNA methylation signatures in maternal circulation as a non-invasive diagnostic method of adverse outcomes of pregnancy [17]. In this study we found that in the first/early second trimester of pregnancy women with GDM have a higher amount of placental DNA that was methylated. In addition to DNA methylation, groups of investigators have examined panels of proteins detected by proteomics that are specific to EVs from GDM pregnancies [25, 26]. Most of these GDM specific proteins carried within circulating EVs were related to metabolism or inflammation as well. Others have focused on the two-fold amounts of circulating EVs released from the placenta in GDM versus normal pregnancies as a biomarker of the disorder [12, 27]. In separate studies, two circulating cell-free miRNAs were detected in the first trimester of GDM subjects [28]. In comparison to cell free DNA/RNA, the transcriptomic cargo within EVs are protected by their stable lipid bilayer and are influenced by their cell of origin and the environment, such as hypoxia and hyperglycemia [29, 30]. Consequently, we sought to investigate non-coding transcripts, i.e. the miRNA cargo of circulating EVs in pregnancy and their potential as biomarkers for adverse pregnancy outcomes.

EVs serve as a basic mode of communication at the cellular level and can regulate various pathways at transcriptional, post-transcriptional and translational levels by cell-to-cell communication, therefore delivering their content, such as microRNAs. Circulating EVs within maternal blood have been shown to have an important role in normal pregnancy and are involved in mediating the pathophysiology of certain pregnancy complications, including GDM [11, 12]. Our study demonstrated differences in the abundance of miRNAs at all gestational trimesters between GDM and normal pregnancy. We identified differences in miRNA expression throughout gestation in EVs isolated from women diagnosed with GDM.

The placenta secretes EVs, via syncytiotrophoblast cells [31, 32], into the maternal circulation from week 6 of gestation [5]. During pregnancy the placenta expresses a unique repertoire of miRNAs which are regulated throughout gestation [33]. This includes trophoblast cells at the maternal-fetal interface which produce a unique expression of miRNAs, including the chromosome 19 miRNA cluster (C19MC) and chromosome 14 miRNA cluster (C14MC) [32, 34]. C19MCs are expressed exclusively from trophoblast cells and can be detected in the peripheral circulation within 2 weeks of implantation [35]. By comparing miRNA expression between EVs from pregnancy against non-pregnant controls we were able to determine that a large number of miRNAs were pregnancy specific, and a significant number were from the C19 or C14MC and therefore of placental origin. Our results aligned with previous reports demonstrating that C19MC are more constitutively expressed throughout gestation, with an increase at term, whereas C14MC have increased expression in early gestation [34, 36]. Expression of hsa-miR-519d-3p, part of the C19MC was constitutively expressed throughout gestation, and has potential roles in immune tolerance during pregnancy [37].

At term around 12–25% of EVs circulating in maternal plasma during pregnancy originate from the placenta [38]. Our study demonstrates a correlation between the expression of miRNAs in the placenta and within maternal circulating EVs during the third trimester and at term around delivery, suggesting that miRNAs in the placenta are packaged within EVs and released into the maternal circulation. Studies have isolated placental-specific EVs circulating in maternal blood, utilizing the membrane protein placental alkaline phosphatase (PLAP) [39] which is specific to syncytiotrophoblast cells of the placenta, and have demonstrated an increased concentration of placental-derived EVs temporally across gestation [5]. However, there is a paucity of knowledge regarding the transcriptomic cargo of placental-specific EVs in the maternal circulation and the changes that occur with complications of pregnancy. Future studies are needed in isolating and investigating the transcriptomic cargo of placental derived EVs throughout pregnancy in normal pregnancy and GDM.

In the first trimester of pregnancy there was distinct expression of miRNAs in EVs isolated from women who went on to be subsequently diagnosed with GDM, compared to women with normal healthy pregnancies. Focusing on miRNAs in the first trimester of pregnancy we are able to observe changes in miRNAs prior to clinical diagnosis, suggesting that these miRNAs are involved in the pathogenesis of the disease rather than due to confounding factors secondary to the disease (metabolic) itself or GDM treatments (insulin or insulin-sensitizing drugs) which may influence the miRNA content of circulating EVs.

Differential expression of miRNAs within EVs in the maternal circulation has previously been investigated in a range of studies [8, 40]. Differences observed in miRNA differential expression between studies may be due to different starting material (serum/plasma/urine) [41] and/or the difference in EV isolation methods. Currently the guidelines on EV isolation methods developed by the International Society for Extracellular Vesicles (ISEV) settled initially on the existing differential centrifugation method [10, 42], with a subsequent call for improving and developing newer isolation methods [42]. This led to the development of a wide variety of isolation and enrichment methods to overcome the heterogeneity of EVs leading to isolation of different EV populations between studies. Enrichment of EVs from biological specimens is a balance between isolation yield and purity, dependent on the method utilized and the experimental question being asked [10]. We employed a precipitation technique to overcome the limitation of small sample amounts to achieve the appropriate ratio of EV yield and purity for this study. In addition, we considered the future implications of such an isolation method, which would be practical and less time consuming, providing the subsequent possibility of easy adaptation in clinical laboratories with a scalable feature.

Within our study we identified a number of novel miRNAs with differential expression in the first trimester of GDM pregnancies, including has-miR-92a-3p, miR-192-5p and miR-451a. Our present study employed stringent cut-off parameters of padjusted/FDR at a log-2-fold difference to our analyses compared to that adapted by Nair et al in a prior study [8], thereby detecting differences in GDM versus a normal pregnancy under stricter criteria. Nair et al [8] with an identification at a less stringent cut-off for detecting a difference in miRNAs at a p<0.05 alone inevitably also found increased expression of miR-92a-3p in EVs isolated from maternal plasma in mid-gestation, and expression was associated with genes targeting pathways associated with diabetes and hyperglycemia. miR-92a-3p regulates insulin biosynthesis [43], and has a role in insulin secretion by pancreatic β cells [44], and in insulin stimulated glucose uptake by skeletal muscle [8]. MiR-192-5p appears to be involved in type 2 diabetes (T2DM) by regulating the expression of several genes involved in T2DM, such as procollagen C-endopeptidase enhancer 2 (PCOLCE2), which is significantly decreased in T2DM [45, 46]. In healthy uncomplicated pregnancies miR-451a is constitutively expressed [41] however, our study found upregulation in the first trimester of pregnancies that went on to be diagnosed with GDM. miR-122 was found to be upregulated in a study by Gillet et al [15] in which the miRNA profile of total EVs from serum in early pregnancy was determined. Upregulation of miR-122 was associated with genes for regulation of glucose homeostasis and insulin secretion. We speculate that dysregulation of EV encapsulated miRNAs in early gestation contributes to the pathogenesis and subsequent development of GDM. To explore this further we went on to investigate the interaction of differentially expressed miRNAs and their target gene pathways.

We observed differential abundance of miR-100-5p, miR-125a-3p, miR-222-3p and miR-92a-3p which target genes in the FOXO signaling pathway. Insulin resistance observed in GDM causes high levels of reactive oxygen species with subsequent oxidative stress. The FOXO family of transcription factors play an important role in regulation of cellular oxidative stress response. They also play a key role in promoting the expression of glucogenic enzymes [47]. We observed enrichment of various such pathways, *i*.*e*. PI3k-Akt Signaling and C28 dependent PI3k-Akt Signaling pathways (targeted by miR-100-5p, miR-125a-3p, miR-149-5p, miR-222-3p and miR-92a-3p), and Th-17 cell differentiation pathways targeted by miR-125a-3p. PI3k-Akt Signaling pathways play a role in cell metabolism, growth, proliferation, and survival. Circulating EVs are thought to contribute to maternal systemic inflammation observed during pregnancy, with an increase in inflammation in obese women, a major risk factor for GDM [38]. miR-92b-3p although not considered specific for pregnancy, another study also detected differential expression of miR-92a-3p and miR-92b-3p at an early gestational stage proposing it as a potential predictor of GDM [8]. miR-92a-3p is involved in a variety of biological functions such as phosphorylation, cell proliferation, differentiation and apoptosis [48]. GDM women post-delivery during the postpartum period expressed higher serum miR-100-5p concentrations [49], while others demonstrated an association with type 1 diabetes mellitus [50]. Our current analysis demonstrated miR-100-5p targeting mRNAs belonging to the PI3K-Akt, NF-κB, MAPK pathways, VEGFR2 mediated vascular permeability pathway and Ubiquitin mediated proteolysis. In addition miR-125a was noted to be overexpressed in insulin responsive tissues while regulating the MAPK pathway genes [51]. Our analysis demonstrated the involvement of miR-125a in epigenetic regulation of gene expression, cellular senescence and second messenger mediated intracellular signaling. Altogether, our results in the context of others, suggest that the miRNA cargo within circulating EVs may be communicating with other maternal organs/cell types and interacting with signaling pathways involved in metabolism and inflammation, which could be influencing the maternal metabolic adaptations observed in women who develop GDM.

Currently testing for GDM begins in the late second trimester as there are no reliable early gestation diagnostics. Therefore, in this study we used miRNA expression data from the first trimester of pregnancy, prior to clinical diagnoses, to identify probable candidate biomarkers for the early detection of GDM. As our study had the limitation of a small sample size, due to the nature of the prospective study design, we built a logistic regression model and trained the model using leave-one-out cross-validation (LOOCV). We found the model had ~90% accuracy during the first trimester in predicting a subsequent GDM outcome employing not a single gene, but rather a panel of genes identified by two different methods, one including all genes expressed in GDM subjects and the other including only the differentially expressed genes in GDM beyond a normal pregnancy (has-miR-92a-3p, hsa-miR-122-5p, hsa-miR-192-5p and hsa-miR-451a or has-miR-92a-3p, hsa-miR-92b-3p, hsa-miR-100-5p and hsa-miR-125a-3p). This is a promising initial step in finding a robust and accurate biomarker panel that performs much better than one single feature in predicting GDM during early pregnancy. To validate our initial predictive model conducted on a small sample size, we tested it further in a previously reported separate cohort consisting of 8 normal and 9 GDM pregnancies at the 2^nd^ trimester [8]. Our predictive model performed well in this validation cohort as well, although not as well as in the 1^st^ trimester of our present study. However, additional studies are required to continue validation of these initial results in a separate prospectively recruited cohort of women with a larger sample size, towards confirming the accuracy of prediction by a panel of genes rather than a single gene or biomarker, or existing methods of using fasting or random glucose and HbA1C concentrations. Only then, can one surmise whether predicting GDM is beneficial, poses further risk, or makes no difference to the mother and baby outcomes.

In summary, our study establishes that the miRNA content of circulating EVs during pregnancy is dependent on gestation and pregnancies with adverse outcomes have a distinct EV miRNA profile. We demonstrate that EV encapsulated panel of miRNAs has the potential to serve as a noninvasive biomarker detecting GDM in early gestation. The placenta acts as a communicator between maternal and fetal compartments and our results show that circulating EVs in pregnancy contain miRNAs packaged from the placenta. Future studies are required to investigate further the mechanistic role of circulating EVs during pregnancy, their pathogenesis role in complications of pregnancy and the utilization of EV associated miRNAs as noninvasive predictors of disease in pregnancy.

## Supporting information

S1 Fig Characterization of EVs. Flow cytometry analysis detecting FITC-labeled PLAP specific EVs (green) represented in left and middle panels with the peak shown in the right panel (A). Representative immunoblot of PLAP from the placenta and HepG2 cell lysate (negative control) demonstrating specificity of the antibody (B).
(PDF)

S2 Fig Distribution of placental-specific miRNAs in early gestation. Boxplots show the distribution of (A) C19MC and (B) C14MC miRNAs from EVs isolated from non-pregnant (NP), normal pregnancy (NORM) and gestational diabetes (GDM) group maternal plasma samples in the first trimester of pregnancy. The significance of the difference between groups was calculated using Kruskal–Wallis one-way analysis of variance followed by post-hoc analysis using Dunn’s test. Bonferroni corrected p-values are shown in the inter-group comparisons with significant differences.
(PDF)

S3 Fig Correlation between miRNAs from the placenta at term and EV-derived miRNAs isolated from maternal plasma at 3^rd^ Trimester in (A) normal pregnancy and (B) GDM. (PDF)

S4 Fig Expression pattern of DE miRs across the three trimesters. Heatmap showing expression pattern of miRNAs which were differentially abundant in EVs across the three trimesters when normal (NORM) (left panel) or GDM (right panel) pregnancies were compared to the non-pregnant samples.
(PDF)

S1 Raw image Original images for immunoblots. (PDF)

S1 Table List of differentially abundant miRNAs between NP and NORM samples. (XLSX)

S2 Table List of differentially abundant miRNAs from EVs between gestational diabetes mellitus (GDM) and normal (NORM) samples. (XLSX)

S3 Table List of differentially expressed miRNAs from placenta between gestational diabetes mellitus (GDM) and normal (NORM) samples. (XLSX)

S4 Table miRNA targets for differentially abundant miRNAs between gestational diabetes mellitus (GDM) and normal (NORM) pregnancy. (XLSX)
