# Supplementary figures and images for: Circulating extracellular vesicles exhibit a differential miRNA profile in gestational diabetes mellitus pregnancies
Source: PLoS One. 2022 May 25;17(5):e0267564. doi: 10.1371/journal.pone.0267564 (PMC9132306; doi:10.1371/journal.pone.0267564)

# S1 Fig

**A**

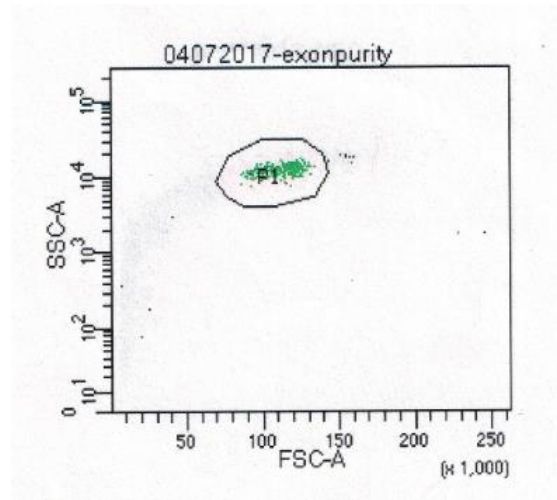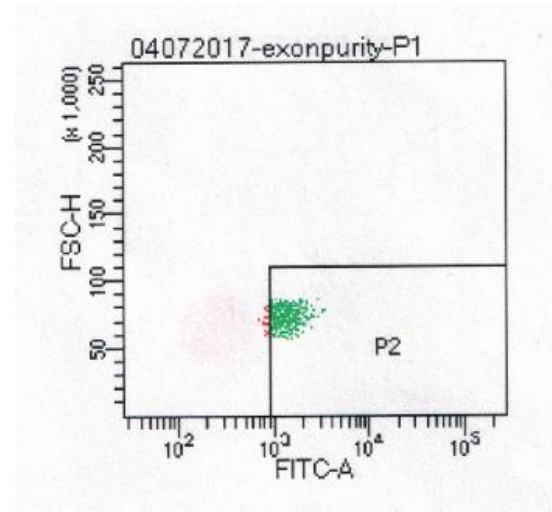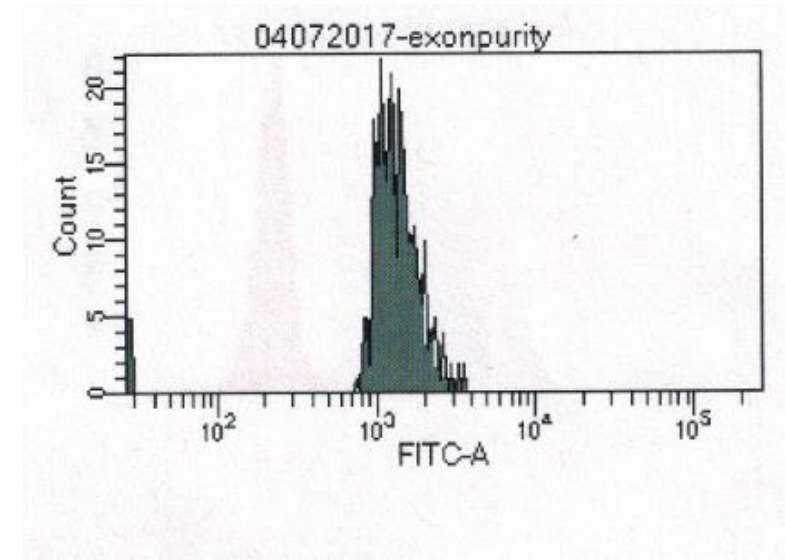

**B**

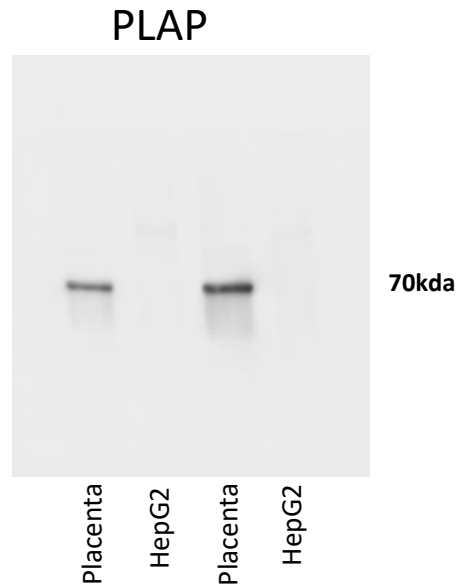

Supplement: S1 Fig — Flow cytometry analysis detecting FITC-labeled PLAP specific EVs (green) represented in left and middle panels with the peak shown in the right panel (A). Representative immunoblot of PLAP from the placenta and HepG2 cell lysate (negative control) demonstrating specificity of the antibody (B). (PDF) [file pone.0267564.s001.pdf]

S2 Fig

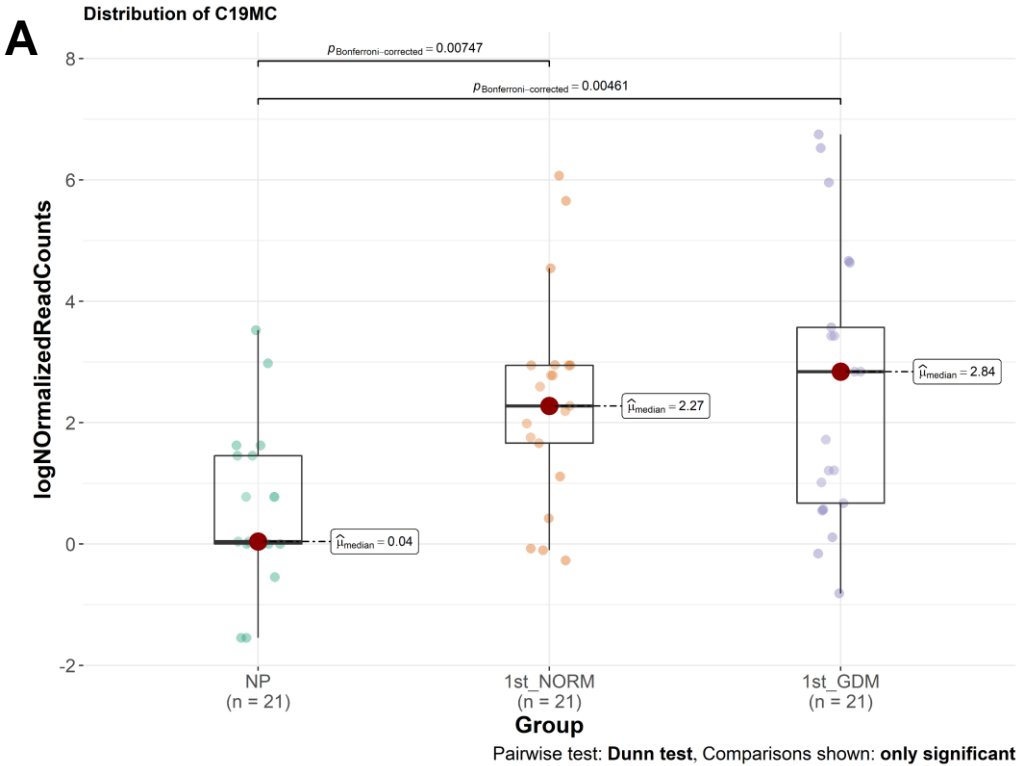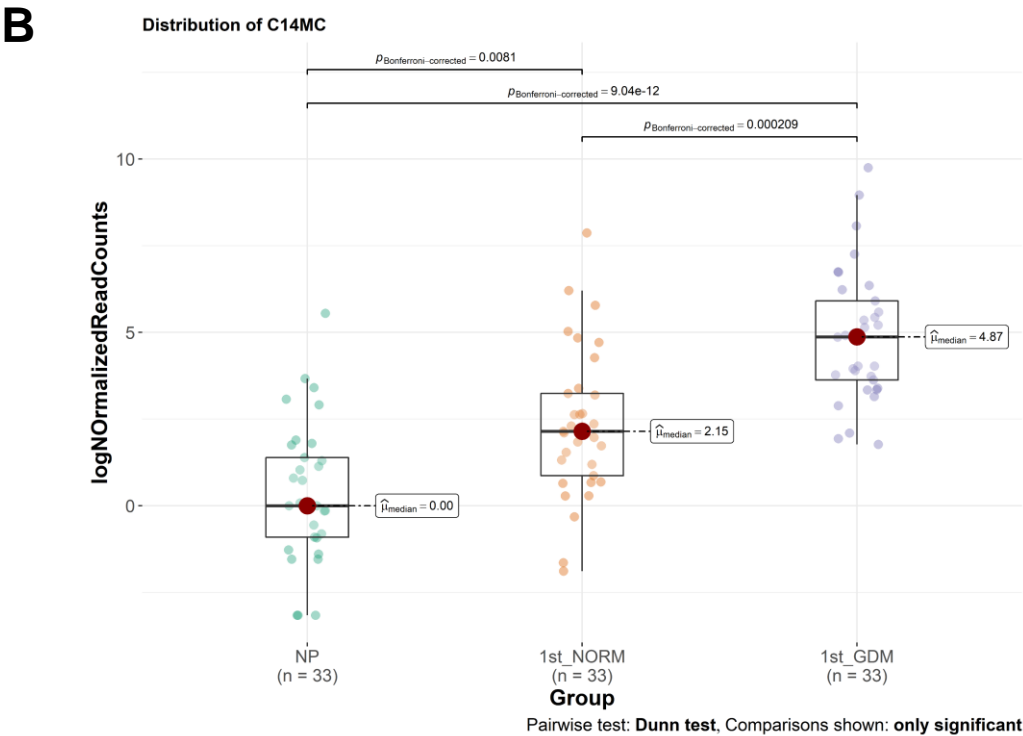

Supplement: S2 Fig — Boxplots show the distribution of (A) C19MC and (B) C14MC miRNAs from EVs isolated from non-pregnant (NP), normal pregnancy (NORM) and gestational diabetes (GDM) group maternal plasma samples in the first trimester of pregnancy. The significance of the difference between groups was calculated using Kruskal–Wallis one-way analysis of variance followed by post-hoc analysis using Dunn’s test. Bonferroni corrected p-values are shown in the inter-group comparisons with significant differences. (PDF) [file pone.0267564.s002.pdf]

S3 Fig

A

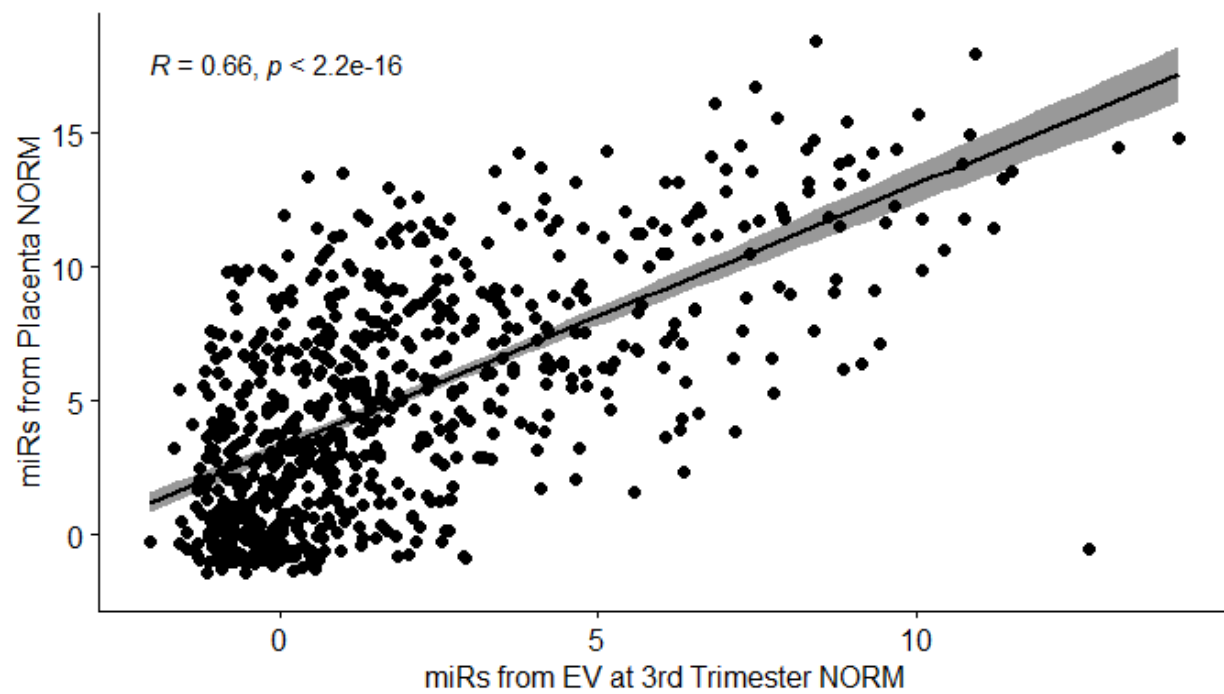

B

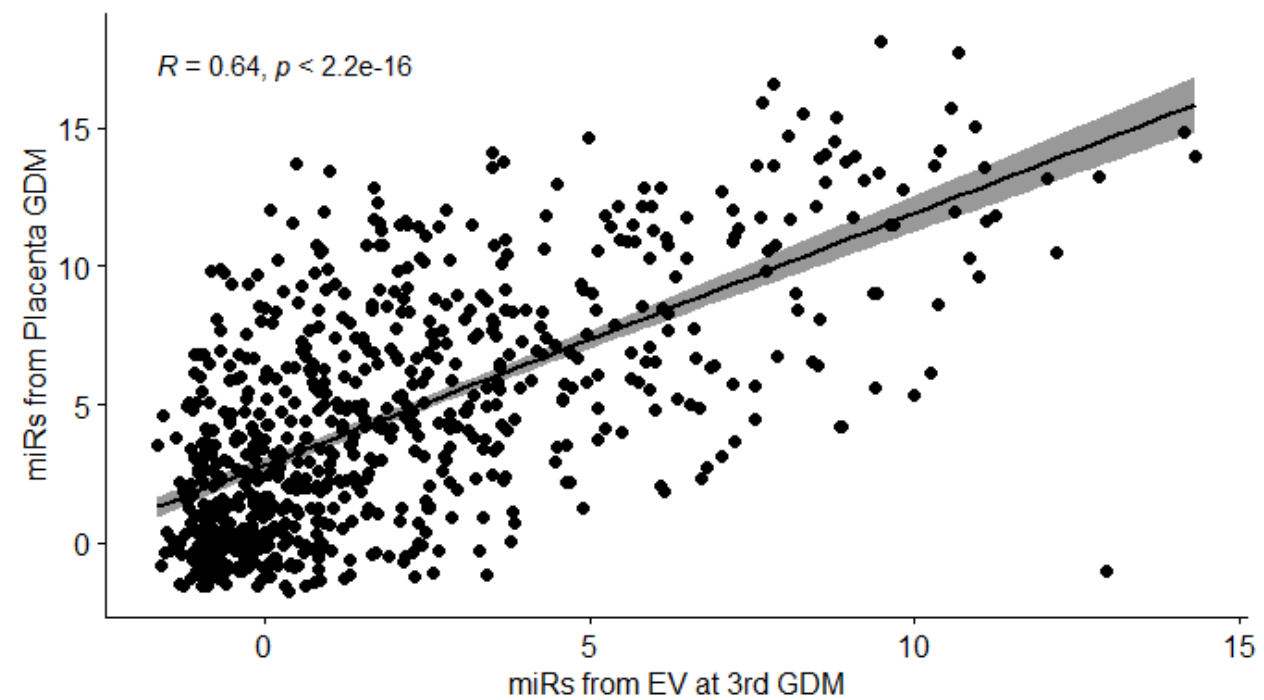

Supplement: S3 Fig — (PDF) [file pone.0267564.s003.pdf]

S4 Fig

Expression pattern of DE miRs across the trimester

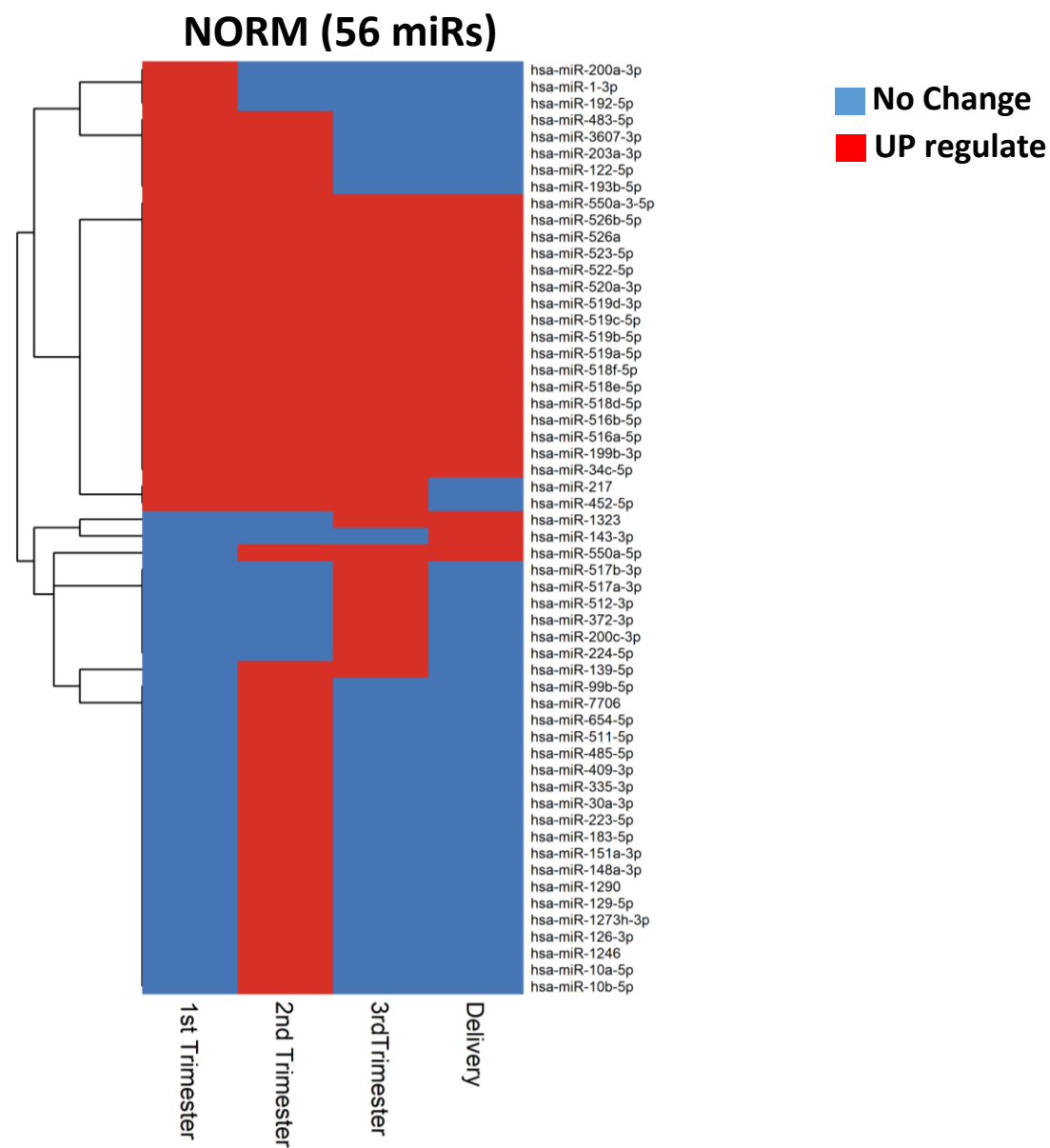

**GDM (311 miRs)**

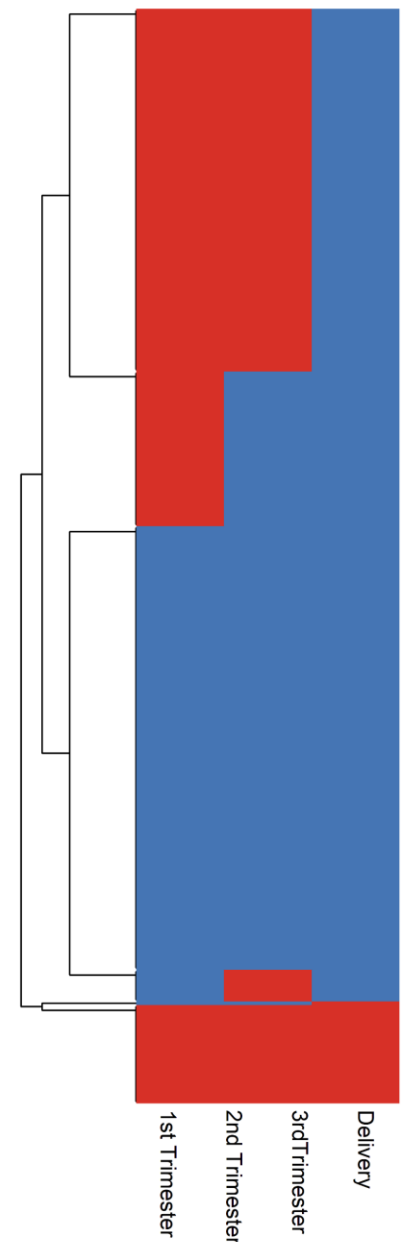

Supplement: S4 Fig — Heatmap showing expression pattern of miRNAs which were differentially abundant in EVs across the three trimesters when normal (NORM) (left panel) or GDM (right panel) pregnancies were compared to the non-pregnant samples. (PDF) [file pone.0267564.s004.pdf]
