# Supplementary material for: Circulating extracellular vesicles exhibit a differential miRNA profile in gestational diabetes mellitus pregnancies
Source: PLoS One. 2022 May 25;17(5):e0267564. doi: 10.1371/journal.pone.0267564 (PMC9132306; doi:10.1371/journal.pone.0267564)
Supplement: S1 Raw image — (PDF) [file pone.0267564.s005.pdf]

S5 raw images immunoblot

CD63

1 2 3

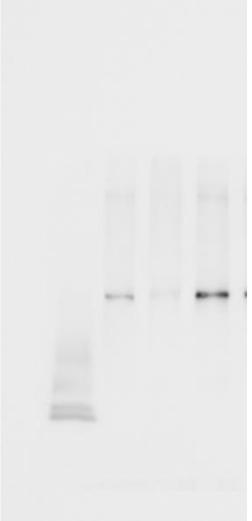

1.Plasma
2.Supernatant
3. EVs

PLAP

1 2 3 4.

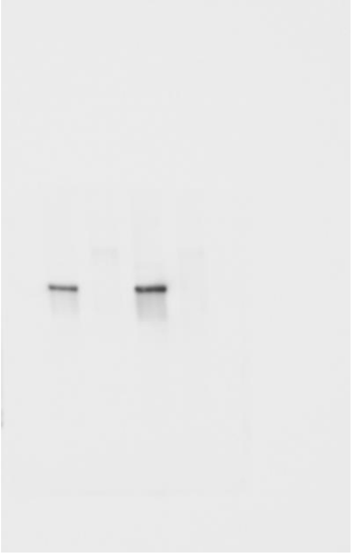

1.Placenta
2.HEFG2 cell lysate
3.Placenta
4.HEFG2 cell lysate

Calnexin

1 2 3 4

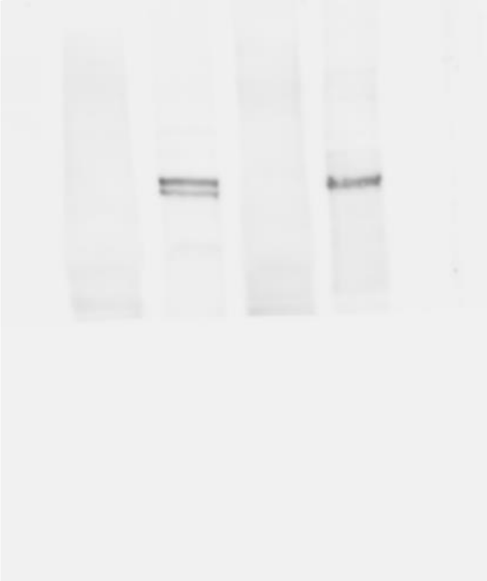

1.Plasma
2.Placenta
3.EVs
4.HEFG2 cell lysate

Flottilin

1 2

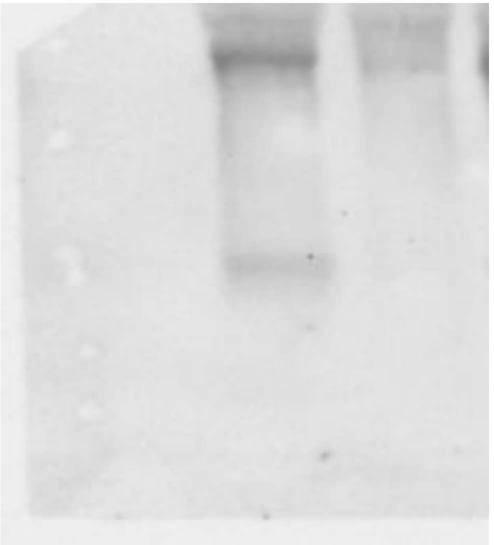

1.EVs
2. Supernatant

S5 raw images immunoblot

CD63

1 2

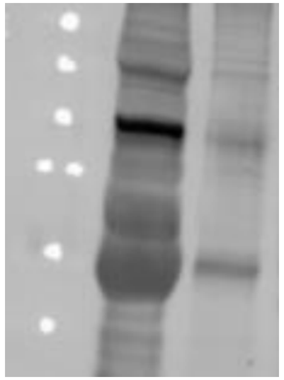

1. EVs

2. placenta

PLAP

1 2

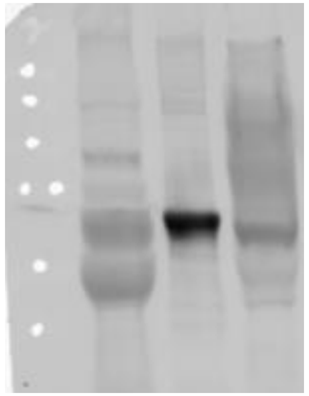

1.placenta
2. EVs
